# Editorial: Diagnostic and therapeutic applications of visible and near-infrared light for the retina

**DOI:** 10.3389/fopht.2025.1743468

**Published:** 2025-12-18

**Authors:** Małgorzata B. Różanowska, Jennifer J. Hunter

**Affiliations:** 1 Cardiff Institute of Tissue Engineering and Repair, Cardiff University, Wales, United Kingdom; 2 School of Optometry and Vision Sciences, Cardiff University, Cardiff, Wales, United Kingdom; 3 School of Optometry and Vision Science, University of Waterloo, Waterloo, ON, Canada

**Keywords:** adaptive optics scanning light ophthalmoscopy (AOSLO), light-induced retinal injury, ophthalmic imaging techniques, optical coherence tomography (OCT), optoretinography, photobiomodulation

Visible and near infrared (NIR) light is utilised in many applications to help diagnose, monitor progression, and/or treat various diseases and retinal conditions. Diagnostic imaging techniques include optical coherence tomography (OCT) and scanning laser ophthalmoscopy (SLO). These rely on visible or near-infrared (NIR) light reflected from the structures of the retina because of variations in their refractive indices across different cellular layers. SLO is a point-scanning system, similar to a confocal microscope, that provides *en face* images of the structures at the back of the eye. Typically, it uses a laser to provide monochromatic exposure to visible and NIR wavelengths ranging from 488 nm to 830 nm, and can be combined with many different imaging modalities, including fluorescence (1–3). In OCT, reflected light of the bandwidths of 40 to 150 nm, typically in 750-900 nm or 1000–1100 nm range, interacts with a reference beam, creating interference patterns that can be decoded into the amplitude and retinal depth of the reflected light, revealing retinal structure at an axial resolution of a few micrometres (4–6).

In this Research Topic, Allegrini et al. described using OCT and NIR reflectance funduscopy to monitor the progression of tangential traction associated with epiretinal membranes (ERMs). Fundus tracking was employed to co-localise the OCT and reflectance images, from which 10 vessel crossings were manually selected. These vessel crossings were then followed up during subsequent examinations, which took place one week before and one month after the peeling surgery. OCT B-scans were used to segment the ERM and the retinal pigment epithelium (RPE), and to determine the vertical projections of each vessel crossing onto the ERM/inner limiting membrane (ILM) and the RPE. The vertical projection of the vessel crossing onto the RPE, as recorded one month after surgery, served as a reference point for measuring the horizontal shift of crossings, as determined by the B-scan in the previous examinations – this is defined as the relaxation index (RI). Measurements performed on 9 eyes of 9 patients demonstrated that there was a statistically significant increase in RI between the initial examination and the examination one week before the surgery, and that there was a statistically significant decrease in RI between the examinations before the surgery and those taking place one month post-surgery, suggesting that RI can be used as an objective parameter to measure the ERM traction and relaxation after surgical removal.

Intensity-based optoretinography (iORG) measures changes in the amplitude of back-scattered/reflected light from photoreceptors after a light stimulus (7–9). Gaffney et al. used iORG with adaptive optics SLO (AOSLO) and microperimetry to investigate the structural and functional changes in cones in 7 retinas affected by retinitis pigmentosa (RP), comparing them with 15 healthy retinas. Using imaging with a low coherence superluminescent diode as the light source, which emitted in the NIR spectrum, the authors determined that the nearest neighbour distance (NND) of cones was largest in patients with RP across different retinal eccentricities. iORG amplitudes recorded in response to photoexcitation with 66-ms pulses of visible light showed that the majority of cones in RP patients demonstrated decreased amplitudes compared with cones in healthy retinas. Using OCT, they recorded longitudinal reflectivity profiles (LRPs), from which the length of the cone outer segment was determined to be shorter in RP cones than in normal cones. Importantly, it was demonstrated that iORG is more sensitive than microperimetry in the detection of functional deficits in cones at the early stages of RP.

Visible and NIR light is also employed for various therapeutic purposes, which include photobiomodulation (PBM). Using narrow-band light (usually within the 600–1100 nm range), PBM targets endogenous chromophores to improve some aspects of retinal health and function. Valter et al. provided a brief overview of preclinical studies, clinical case studies, and clinical trials that tested PBM for different retinal and other ocular conditions, including retinopathy of prematurity, RP, Stargardt’s disease, Leber’s hereditary optic neuropathy, diabetic retinopathy, diabetic macular oedema, age-related macular degeneration, amblyopia, myopia, corneal trauma, keratitis, and hydroxyapatite orbital implant exposure. The safety aspects were discussed briefly, primarily in terms of safety for tissues other than the retina. However, in the majority of reports from clinical trials, the exposure parameters provided in publications are not sufficient to evaluate retinal irradiance and compare radiant exposure with the safety thresholds established for ophthalmic devices with respect to retinal susceptibility to light-induced injury (10). A device used for the treatment of myopia, which emits a 654 nm wavelength with a power of 0.2 mW through an aperture of 7 mm in diameter, was found to exceed the photochemical and photothermal safety limits within 34 and 33 s of exposure, respectively (11, 12). However, children using these devices are instructed to look directly into the beam for 180 s twice a day, 5 days a week, for several years. While the safety aspects of PBM for myopia control have been questioned (11–15), resulting in manufacturers of PBM devices being required to provide thorough safety evaluations, including primate trials, before any further clinical trials can be approved in China (16), the irradiance levels used for the treatment of age-related macular degeneration (AMD) and other retinal conditions are several orders of magnitude greater than those used for myopia, therefore raising serious concerns about their photochemical and photothermal safety for the retina. Table 1, provided by Valter et al., lists clinical trials of PBM for AMD, in which five trials used 5-minute exposures to a combination of 590 nm yellow light of 5 mW/cm^2^ irradiance, 660 nm red light of 65 mW/cm^2^ irradiance and 850 nm NIR light of 8 mW/cm^2^ irradiance. These exposures are highly likely to greatly exceed the retinal safety thresholds for thermal and photochemical injuries, but the evaluation of retinal irradiance from these devices has not yet been reported. It is worth considering that, while several hypothetical scenarios have been proposed to explain the observed beneficial effects of PBM (17, 18), the mechanisms involved remain unclear. Furthermore, there has been no systematic investigation into the optimal wavelength, irradiance, exposure duration, or frequency of repetition. This is important because a recent study indicates that the beneficial effects of PBM on visual acuity in AMD patients can be achieved with ten 10-minute exposures to only 15 µW red light incident on the cornea (19).

Finally, the interaction of light with RPE cells was considered. Denton et al. used mathematical modelling to examine photochemical and photothermal types of damage in relation to pigmented RPE cells *in vitro*. Pope et al. demonstrated *in vitro* that the light exposure that elevates the temperature of RPE cells exacerbates photochemical damage. These studies will have an impact on understanding and predicting damage thresholds and mechanisms in the human retina.
